# Factors associated with chronic musculoskeletal pain in patients with chronic kidney disease

**DOI:** 10.1186/1471-2369-15-6

**Published:** 2014-01-08

**Authors:** Heng-Jung Hsu, Chiung-Hui Yen, Kuang-Hung Hsu, I-Wen Wu, Chin-Chan Lee, Ming-Jui Hung, Chiao-Yin Sun, Chia-Chi Chou, Yung-Chih Chen, Ming-Fang Hsieh, Chun-Yu Chen, Chiao-Ying Hsu, Chi-Jen Tsai, Mai-Szu Wu

**Affiliations:** 1 Department of Nephrology, Chang Gung Memorial Hospital, 222, Mai-Chin Road, Keelung 20401, Taiwan; 2 The Graduate Institute of Clinical Medical Sciences, Chang Gung University Medical College, Taoyuan School of Medicine, Taipei, Taiwan; 3 Chang Gung University, Taipei, Taiwan; 4 Department of Pediatrics and Rheumatology, Taipei Medical University Hospital, Taipei, Taiwan; 5 Laboratory of Epidemiology, Department of Health Care Management, Chang Gung University, Taipei, Taiwan; 6 Department of Cardiology, Chang Gung Memorial Hospital, Keelung, Taiwan; 7 Division of Nephrology, Taipei Medical University Hospital, Taipei, Taiwan; 8 Department of Internal Medicine, Taipei Medical University, Taipei, Taiwan

**Keywords:** Chronic pain, Musculoskeletal pain, Chronic kidney disease, Hyperuricemia

## Abstract

**Background:**

Chronic musculoskeletal (MS) pain is common in patients with chronic kidney disease (CKD) undergoing haemodialysis. However, epidemiological data for chronic MS pain and factors associated with chronic MS pain in patients with early- or late-stage CKD who are not undergoing dialysis are limited.

**Method:**

A cross-sectional study to evaluate the prevalence of chronic MS pain and factors associated with chronic MS pain in patients with early- and late-stage CKD who were not undergoing dialysis, was conducted. In addition, the distribution of pain severity among patients with different stages of CKD was evaluated.

**Results:**

Of the 456 CKD patients studied, 53.3% (n = 243/456) had chronic MS pain. Chronic MS pain was independently and significantly associated with hyperuricemia as co-morbidity, as well as with the calcium × phosphate product levels. In CKD patients with hyperuricemia, chronic MS pain showed a negative, independent significant association with diabetes mellitus as a co-morbidity (odds ratio: 0.413, p = 0.020). However, in the CKD patients without hyperuricemia as a co-morbidity, chronic MS pain showed an independent significant association with the calcium × phosphate product levels (odds ratio: 1.093, p = 0.027). Furthermore, stage-5 CKD patients seemed to experience more severe chronic MS pain than patients with other stages of CKD.

**Conclusion:**

Chronic MS pain is common in CKD patients. Chronic MS pain was independently and significantly associated with hyperuricemia as co-morbidity, and with the calcium × phosphate product levels in early- and late-stage CKD patients who were not on dialysis.

## Background

The worldwide prevalence of chronic kidney disease (CKD) continues to increase [1]. CKD patients develop many complications, which lead to a high risk of co-morbidities and mortality [2-4].Therefore, two issues are important for healthcare professionals caring for CKD patients: (1) prolonging the life span and (2) improved quality of life of patients.

CKD patients have a poor quality of life [5]. This may be attributed to the underlying disease or to the complications associated to CKD. The Dialysis Outcomes and Practice Patterns Study (DOPPS), a large, international, observational study, demonstrated that quality-of-life indicators from SF-36 were associated with differential survival and morbidity [6]. Body pain is one of the most important qualitative parameters for evaluating patients’ quality of life [7]. To improve quality of care provided to CKD patients, it is important to understand and relieve body pain.

It has been reported that 82% of CKD patients undergoing dialysis had chronic pain, [8] and 35-70% of patients had moderate to severe chronic pain [9]. Chronic pain is common in CKD patients [10], especially in patients with end-stage renal disease (ESRD) [11]. Musculoskeletal (MS) pain is the most common symptom of chronic pain syndromes in ESRD patients [8]. Few studies have focused on chronic pain in early-stage CKD patients.

Despite the understanding of prevalence and risk factors associated with chronic MS pain in CKD patients undergoing dialysis, the epidemiologic data for factors associated with chronic MS pain in early- and late-stage CKD patients who are not on dialysis, are limited. In addition, information about the prevalence of non-steroidal anti-inflammatory drugs (NSAID) or Chinese herbs used for pain relief in CKD patients is also limited. The aims of this study were to determine the prevalence of chronic MS pain in CKD patients and to identify the factors associated with chronic MS pain.

## Methods

### Patients

**Figure 1:**
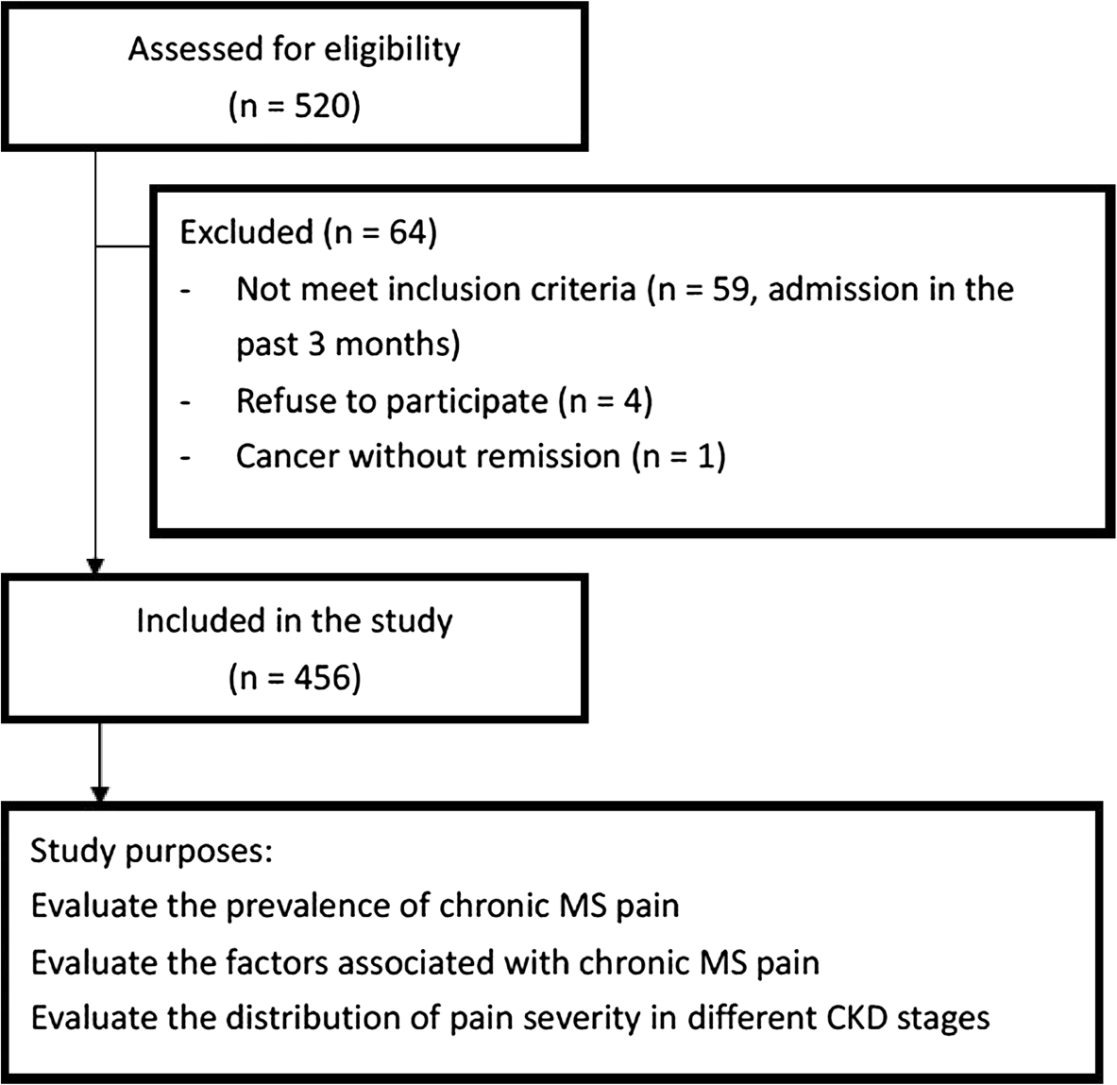
Flow chart indicates patient enrolment.

Pre-dialysis CKD patients who visited an outpatient clinic at the Nephrology Department of Chang Gung Memorial Hospital at Keelung, from March 2006 to July 2007, were recruited for the study. Patients satisfying the following criteria were included in this study consecutively: between 18 to 80 years old, and with no spontaneous improvement or progression of renal disease in the 3 months prior to the start of the study. Patients with acute illness requiring hospital admission in the past 3 months, cancer without remission, or who were unwilling to participate in the trial were excluded from the study. CKD was defined as the presence of persistent proteinuria, or a decreased estimated glomerular filtration rate (eGFR) of <90 mL/min per 1.73 m^2^ [determined by the CKD Epidemiology Collaboration (CKD-EPI) creatinine equation], in two separate measurements within an interval of 3 months [12]. In accordance with the National Kidney Foundation/Dialysis Outcomes Quality Initiative (NKF/DOQI) classification system, these patients were classified into stages 1, 2, 3, 4, or 5 for descriptive purposes. A total of 456 patients who provided informed consent were enrolled into the study (Figure 1). This study complies with the Declaration of Helsinki and was approved by the Ethics Committee of the Institutional Review Board at Chang Gung Memorial Hospital. The study was conducted at the CKD centre of the Chang Gung Memorial Hospital, Keelung, Taiwan.

### Study design

All eligible patients were carefully interviewed to identify primary disease and current medications. At the time of the visit, we also checked the patients’ blood pressure and performed laboratory tests. Chronic MS pain was evaluated by the Community Oriented Program for the Control of Rheumatic Diseases (COPCORD) questionnaire [13]. For patients with MS pain, pain was assessed using a visual analogue scale (VAS), with a range of 0 mm (no pain) to 100 mm (very painful), after which the patients were examined by a rheumatologist to confirm the information and characterize MS pain symptoms. For the calculation of prevalence and for all other analyses, chronic MS pain was defined as non-traumatic MS pain for a VAS score of >1 for more than 3 months [8]. Pain severity categories were defined in this study as follows: VAS pain score of 30 mm or less, mild pain; VAS pain score of 31 mm to 69 mm, moderate pain; and VAS pain score of 70 mm or more, severe pain. This categorization is based, in part, on the findings of Collins et al. [14].

Basic demographic data were collected, including information on age, gender, nutrition status (body mass index [BMI] and waist circumference), history of smoking, alcohol and betel nut consumption, current use of NSAIDs or Chinese herbal medicines, and the presence of diabetes, hypertension, coronary artery disease (CAD), peripheral arterial disease (PAD), congestive heart failure (CHF), stroke, hyperuricemia, systemic lupus erythematosus (SLE), urolithiasis, polycystic kidney disease (PKD), rheumatoid arthritis (RA), spondylarthropathies, spine osteoarthritis (OA), and back pain. Current use of diuretics or allopurinol was also collected. Baseline haematological and biochemical data of these patients were also collected. Smoking was defined as the consumption of more than 10 cigarettes per day for at least one year. Alcohol use was defined as daily consumption of more than 80 mg of alcohol/day for more than one year. Current use of NSAIDs was defined by self-reported use of ibuprofen, naproxen, sulindac, piroxicam, indomethacin, tolmetin, or diclofenac (with brand names and combination formulas identified) daily or nearly every day, for the last 30 days. Current use of Chinese herbs was defined by self-reported use of any Chinese herb daily or nearly every day, for the last 30 days. Diabetes mellitus was defined as a fasting glucose level ≥126 mg/dL or use of any hypoglycaemic medication. Hypertension was considered to be present if the patient was on antihypertensive medicine, or if blood pressure was >140/90 mm Hg. The presence of CAD was confirmed by coronary angiography, and CHF was diagnosed on the basis of the Framingham criteria for heart failure [15]. PAD was defined based on either the results of peripheral Doppler analysis, or a history of amputation. Stroke was diagnosed by clinical symptoms and the results of imaging studies. Hyperuricemia was considered if the patient was on medicine for uric acid control, or if blood uric acid level was >8.0 mg/dL. SLE was defined as per the eleven criteria by the American Rheumatism Association. The presence of urolithiasis was defined by kidney echo report or previous history of renal stone removal. The presence of PKD was defined by kidney echo report. The presence of RA was defined by the 2010 ACR/EULAR Rheumatoid Arthritis Classification Criteria [16]. The presence of spondylarthropathy was defined by the European Spondylarthropathy Study Group criteria for spondylarthropathy [17]. Spine OA was defined by clinical history, findings on physical examination, and radiographic findings suggested by rheumatologists.

### Laboratory measurements

Full laboratory profiles were obtained. The laboratory parameters included levels of blood urea nitrogen, serum creatinine (Scr), haemoglobin, albumin, calcium (Ca), phosphate (P), intact parathyroid hormone (iPTH), total cholesterol, triglycerides, uric acid, and high-sensitivity C-reactive protein (hs-CRP). Serum creatinine levels were assessed by spectrophotometric analysis using a modified kinetic Jaffe reaction.

### Statistical analysis

Descriptive statistics were expressed as means with standard deviation or percentage frequency, as appropriate. All variables were tested for normal distribution by a Kolmogorov-Smirnov test. The Student’s t-test or Mann–Whitney U test was applied to compare means of continuous variables. Categorical data were tested using the chi-square test. Binary logistic regression was appropriately used to test the factors associated with chronic MS pain. To identify independent associations, stepwise multivariate regression analysis was used, and variables including age, gender, and *p* < 0.05 in the univariate analysis were selected. All analyses were performed by using the commercially available statistics software, SPSS version 15.0 for Windows.

## Results

### Characteristics of the study population

Mean age of the patients was 63.3 ± 14.1 years and 42.5% (n = 194/456) of patients were male. The NSAID and Chinese herb use by CKD patients was around 7.8% (n = 40/456), and 9.6% (n = 44/456), respectively. Approximately 30.7% (n = 140/456) of patients had DM as a co-morbidity (Table 1). With respect to CKD stage, a large proportion of patients were stage 1 (28.3%), stage 2 (24.1%), and stage 3 (24.8%) CKD, whereas a small proportion of patients were stage 4 (17.8%) and stage 5 (5%) CKD (Table 2). Among the CKD patients studied, 53.3% (243/456) had chronic MS pain.

**Table 1:** Baseline characteristics classified according to the presence of chronic musculoskeletal (MS) pain

	**All patients n = 456**	**No chronic MS pain n = 213**	**Chronic MS pain n = 243**	**p**
Age (y)	63.3 ± 14.1	62.4 ± 14.5	64.6 ± 13.5	0.09
Male (n, %)	194 (42.5%)	64 (30.0%)	130 (53.5%)	<0.001*
Body mass index (Kg/m^2^)	25.5 ± 3.9	25.0 ± 3.4	26.3 ± 4.3	0.001*
Waist (cm)	89.9 ± 10.9	87.8 ± 10.2	93.2 ± 11.2	0.003*
Smoking (n, %)	181 (39.7%)	89 (41.8%)	92 (37.8%)	0.754
Alcohol use (n, %)	131 (28.7%)	65 (30.5%)	66 (27.2%)	0.990
Betel nut use (n, %)	26 (5.7%)	8 (3.8%)	18 (7.4%)	0.078
NSAID use (n, %)	40 (7.8%)	16 (7.5%)	20 (8.2%)	0.854
Chinese herb use (n, %)	44 (9.6%)	22 (10.3%)	22 (9.1%)	0.822
Co-morbidity				
DM (n, %)	140 (30.7%)	75 (35.2%)	65 (26.7%)	0.034*
Hypertension (n, %)	286 (62.7%)	124 (58.2%)	162 (66.7%)	0.059
CAD (n, %)	35 (7.6%)	19 (8.9%)	16 (6.5%)	0.312
PAD (n, %)	2 (0.4%)	1 (0.5%)	1 (0.4%)	0.935
CHF (n, %)	13 (2.9%)	7 (3.2%)	6 (2.4%)	0.465
Stroke (n, %)	19 (4.2%)	7 (3.2%)	12 (4.9%)	0.429
Hyperuricemia (n, %)	226 (49.6%)	54 (25.5%)	172 (71.0%)	<0.001*
SLE (n, %)	2 (0.4%)	1 (0.5%)	1 (0.4%)	0.935
Urolithiasis (n, %)	15 (3.3%)	8 (3.8%)	7 (2.9%)	0.502
PKD (n, %)	1 (0.2%)	1 (0.5%)	0 (0%)	0.372
RA (n, %)	3 (0.7%)	2 (0.9%)	1 (0.4%)	0.723
Spondylarthropathies (n,%)	0 (0%)	0 (0%)	0 (0%)	1.000
Spine OA (n, %)	3 (0.7%)	0 (0%)	3 (1.2%)	0.372
Back pain (n, %)	48 (10.5%)	1 (0.5%)	48 (19.8%)	<0.001*
Diuretics use (n, %)	63 (13.8%)	27 (12.7%)	36 (14.8%)	0.454
Allopurinol use (n, %)	213 (46.7%)	50 (23.5%)	163 (67.1%)	<0.001*
Blood pressure				
Systolic pressure (mm Hg)	133 ± 19	130 ± 18	135 ± 19	0.006*
Diastolic pressure (mm Hg)	73 ± 10	73 ± 10	74 ± 11	0.631

Notes: Values expressed as mean ± SD or percent.

*Abbreviations*: MS: musculoskeletal, NSAID: non-steroid anti-inflammatory drug, CKD: chronic kidney disease, DM: diabetes mellitus, CAD: coronary artery disease, PAD: peripheral arterial disease, CHF: congestive heart failure, SLE: systemic lupus erythematosus, PKD: polycystic kidney disease, RA: rheumatoid arthritis, OA: osteoarthritis.

*Statistical significance based on the chi-square test for categorical variables, and t test for continuous variables.

**Table 2:** Laboratory parameters classified according to the presence of chronic musculoskeletal (MS) pain

	**All patients n = 456**	**No chronic MS pain n = 213**	**Chronic MS pain n = 243**	**p**
Serum parameters				
BUN (mg/dL)	8.4 ± 3.4	8.6 ± 3.6	8.3 ± 3.1	0.019
Scr (mg/dL)	1.5 ± 1.1	1.4 ± 1.1	1. 6 ± 1.1	0.119
eGFR (mL/min/1.73 m^2^)	65 ± 34	68 ± 36	63 ± 31	0.107
CKD stage				0.074
1 (n, %)	129 (28.3%)	77 (36.2%)	52 (21.4%)	
2 (n, %)	110 (24.1%)	44 (20.7%)	66 (27.2%)	
3 (n, %)	113 (24.8%)	49 (23.0%)	64 (26.3%)	
4 (n, %)	81 (17.8%)	32 (15.0%)	49 (20.2%)	
5 (n, %)	23 (5.0%)	11 (5.2%)	12 (4.9%)	
Haemoglobin (g/dL)	11.8 ± 2.1	11.4 ± 2.1	12.4 ± 2.1	0.010*
Albumin (g/dL)	3.9 ± 0.5	3.9 ± 0.6	3.9 ± 0.5	0.882
Ca (mg/dL)	9.2 ± 0.7	9.3 ± 0.5	9.2 ± 0.8	0.631
P (mg/dL)	3.9 ± 0.8	3.8 ± 0.7	4.0 ± 0.9	0.222
Ca × P (mg^2^/mL^2^)	32.3 ± 13.4	30.7 ± 13.5	34.1 ± 13.3	0.039*
iPTH (g/mL)	62.1 ± 55.1	62.2 ± 54.5	62.0 ± 56.0	0.964
Cholesterol (mg/dL)	193.6 ± 38.7	196.1 ± 45.8	189.3 ± 23.2	0.586
Triglycerol (mg/dL)	116.2 ± 65.6	120.3 ± 84.0	111.2 ± 35.0	0.699
Uric acid (mg/dL)	5.4 ± 2.1	5.5 ± 2.1	5.3 ± 2.1	0.294
hsCRP (mg/L)	4.6 ± 11.8	4.25 ± 13.3	4.96 ± 9.4	0.535

Notes: Values expressed as mean ± SD or percent.

*Abbreviations*: MS: musculoskeletal, BUN: blood urea nitrogen, eGFR: estimated glomerular filtration rate, Ca: calcium, P: phosphate, Ca × P: product of calcium and phosphate, iPTH: intact parathyroid hormone, hs-CRP: high-sensitivity C reactive protein.

*p < 0.05 (p values are based on Student *t* test for analysis of continuous variables).

### Chronic MS pain and pain severity in different CKD stages

**Figure 2:**
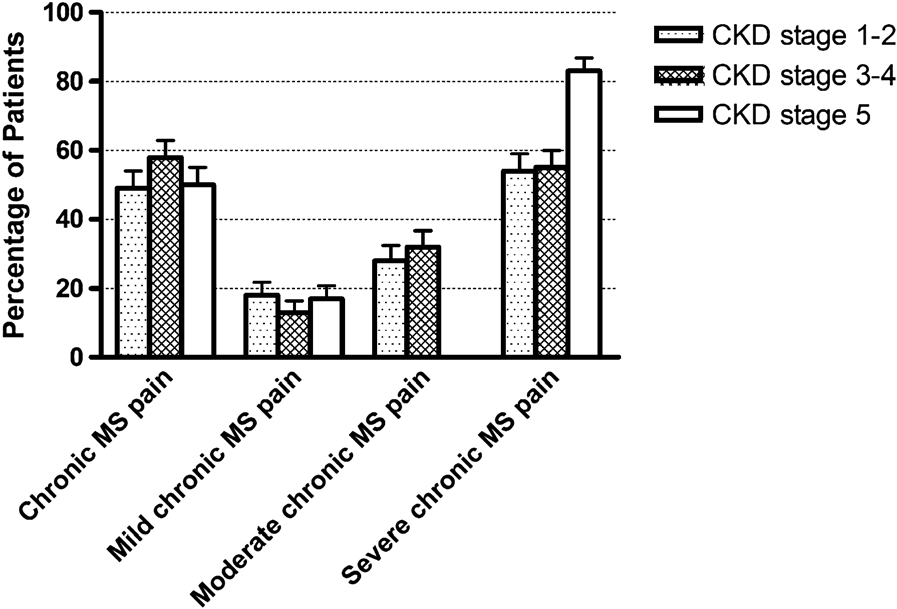
**The percentage of chronic MS pain in CKD patients; the percentage of mild, moderate and severe chronic MS pain in CKD patients with chronic MS pain.** Abbreviations: MS: musculoskeletal, CKD: chronic kidney disease.

CKD patients were divided into three groups: early CKD (CKD stage 1–2), CKD stage 3–4, and CKD stage 5, in order to compare the percentage of patients with chronic MS pain between groups. It was found that similar prevalence of chronic MS pain was noted in early CKD, CKD stage 3–4, and CKD stage 5 [CKD stage 1–2 vs. CKD stage 3–4 vs. CKD stage 5: 49.4% (n = 118/239) vs. 58.2% (n = 113/194) vs. 52.2% (n = 12/23), p = 0.411] (Figure 2).

In CKD patients with chronic MS pain, severe pain accounted for 58% (n = 141/243) of chronic MS pain, moderate pain about 28.4% (n = 69/243), and mild pain only accounted for 15.6% (n = 38/243) of chronic MS pain. Mild chronic MS pain was noted in 17.8% (n = 21/118) of CKD patients with stage 1–2 disease, 13.3% (n = 15/113) of CKD patients with stage 3–4 disease, and 16.7% (n = 2/12) of CKD patients with stage 5 disease (p = 0.594). Moderate chronic MS pain was noted in 28% (n = 33/118) of CKD patients with stage 1–2 disease, 31.9% (n = 36/113) of CKD patients with stage 3–4 disease, and 0% (n = 0/12) of CKD patients with stage 5 disease (p < 0.001). Severe chronic MS pain was found in 54.2% (n = 64/118) of CKD patients with stage 1–2 disease, 54.9% (n = 62/113) of CKD patients with stage 3–4 disease, and 83.3% (n = 10/12) of CKD patients with stage 5 disease (p < 0.001).

### Observed differences between CKD patients with chronic MS pain and no chronic MS pain

**Figure 3:**
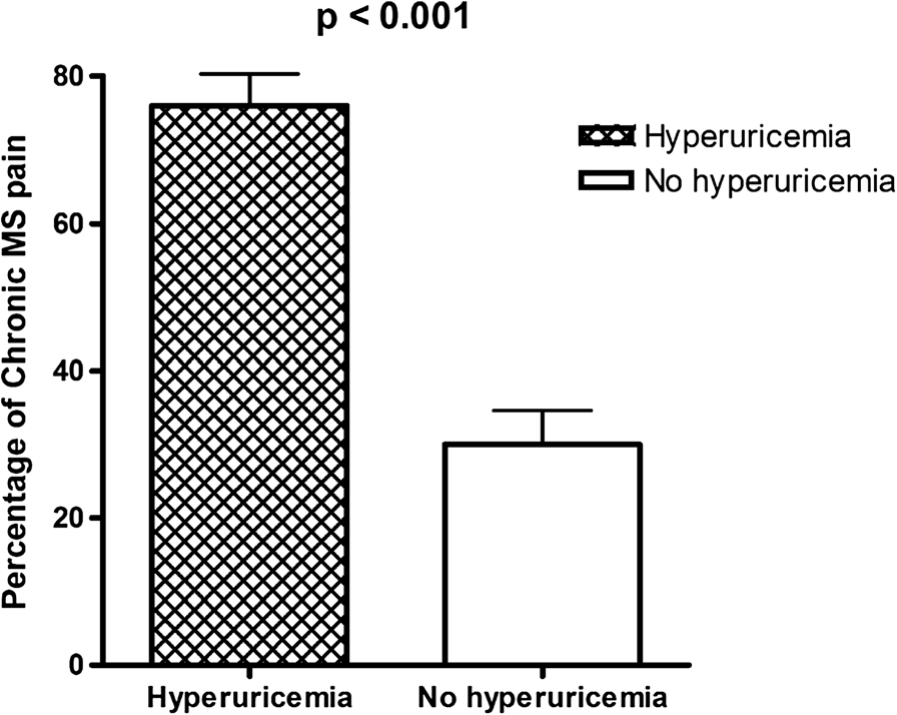
The percentage of chronic MS pain was significantly higher in patients with co-morbidity of hyperuricemia rather than without hyperuricemia (76.1% vs. 30.1%).

Compared with patients who experienced no chronic MS pain, those who reported chronic MS pain were more likely to be male, have a higher BMI and waist circumference, higher systolic blood pressure, and were less likely to have DM as a co-morbidity. In particular, chronic MS pain in CKD was related to hyperuricemia and back pain co-morbidities. One hundred seventy two patients (76.1%, 172/226) who had hyperuricemia had chronic MS pain, but only 71 patients (30.1%, 71/230) without hyperuricemia had chronic MS pain (p < 0.001) (Figure 3). Higher haemoglobin levels (chronic MS pain vs. no chronic MS pain: 12.4 ± 2.1 vs. 11.4 ± 2.1 g/dL, p = 0.010) and Calcium × phosphate product (chronic MS pain vs. no chronic MS pain: 34.1 ± 13.3 vs. 30.7 ± 13.5 mg^2^/mL^2,^ p = 0.039) were also linked with the presence of chronic MS pain. Chronic MS pain was not associated with age, smoking, alcohol/betel nut consumption, or co-morbidities such as hypertension, CAD, PAD, CHF, stroke, SLE, urolithiasis, PKD, RA, spondylarthropathies, spine OA, and diuretics use. In particular, there was no significant difference between current NSAID and Chinese herb use in patients with and without chronic MS pain (NSAID: 8.2% vs. 7.5%, p = 0.854; Chinese herb: 9.1% vs. 10.3%, p = 0.822). The presence of chronic MS pain was also not associated with renal function (BUN and Scr, eGFR, CKD stage), albumin, Ca, P, iPTH, cholesterol, triglycerides, uric acid, or hs-CRP.

### Factors associated with chronic MS pain by binary logistic regression analysis

In this particular study, univariate analysis indicated that chronic MS pain was significantly associated with male gender, higher BMI, co-morbidity of hyperuricemia and back pain, higher systolic blood pressure, higher haemoglobin levels, higher Calcium × phosphate product, and less co-morbidity of DM. However, after multivariate analysis, chronic MS pain was only positively and independently, significantly associated with hyperuricemia and a higher Calcium × phosphate product, whereas male gender, BMI, co-morbidity of back pain and DM, systolic blood pressure, and haemoglobin level lost significance (Table 3). CKD patients were then sub-grouped into patients with or without hyperuricemia as co-morbidity. In CKD patients with hyperuricemia, multivariate analysis indicated that only DM was negatively associated with chronic MS pain (odds ratio: 0.413, p = 0.02), but the calcium × phosphate product lost significance. However, in CKD patients without hyperuricemia, multivariate analysis showed that the calcium × phosphate product was independently and significantly associated with chronic MS pain (odds ratio: 1.093, p = 0.027).

**Table 3:** Factors associated with chronic MS pain: univariate and multivariate analyses

	**Univariate**	**95% confidence interval**	**p**	**Multivariate**	**95% confidence interval**	**p**
	**Odds ratio**			**Odds ratio**		
**All patients**						
Age (y)	1.002	0.985–1.018	0.913			
Male gender	2.295	1.562–3.374	<0.001			
Body mass index (Kg/m^2^)	1.092	1.037–1.151	0.001			
Co-morbidity of DM	0.908	0.603–1.368	0.644			
Co-morbidity of hyperuricemia	7.167	4.655–11.034	<0.001	8.235	2.129–31.852	0.002*
Co-morbidity of back pain	85.808	11.722–628.114	<0.001			
Systolic blood pressure (mmHg)	1.015	1.004–1.026	0.007			
Haemoglobin (g/dL)	1.256	1.050–1.503	0.013			
Ca × P (mg^2^/mL^2^)	1.226	1.044-1.520	0.010	1.028	1.008–1.131	0.022*
**Patients with co-morbidity of hyperuricemia**						
Age (y)	0.985	0.961–1.011	0.258			
Male gender	2.080	0.964–4.486	0.062			
Body mass index (Kg/m^2^)	1.093	0.981–1.194	0.057			
Co-morbidity of DM	0.481	0.235–0.981	0.044	0.413	0.196–0.815	0.020^#^
Co-morbidity of back pain	8.995	0.651–18.143	0.998			
Systolic blood pressure (mmHg)	1.011	0.992–1.031	0.248			
Haemoglobin (g/dL)	1.415	1.016–1.971	0.040			
Ca × P (mg^2^/mL^2^)	0.994	0.937–1.054	0.835			
**Patients without co-morbidity of hyperuricemia**						
Age (y)	1.020	0.983–1.042	0.076			
Male gender	0.958	0.548–1.676	0.881			
Body mass index (Kg/m^2^)	1.027	0.948–1.113	0.515			
Co-morbidity of DM	1.505	0.845–2.679	0.165			
Co-morbidity of back pain	6.182	0.353–17.313	0.998			
Systolic blood pressure (mmHg)	1.013	0.997–1.029	0.107			
Haemoglobin (g/dL)	1.085	0.842–1.398	0.528			
Ca × P (mg^2^/mL^2^)	1.083	1.012–1.159	0.021	1.093	1.010–1.179	0.027^#^

*p from logistic regression adjusted for age, male gender, body mass index, co-morbidity of diabetes, hyperuricemia, and back pain, systolic blood pressure, serum haemoglobin, and product of calcium and phosphate levels.

#p from logistic regression adjusted for age, male gender, body mass index, co-morbidity of diabetes, and back pain, systolic blood pressure, serum haemoglobin, and product of calcium and phosphate levels.

*Abbreviations*: MS, musculoskeletal; DM, diabetes mellitus, CA × P, product of calcium and phosphate.

## Discussion

The mechanisms associated with chronic MS pain in CKD patients are multi-factorial. The present study enrolled mostly early CKD patients, and for the first time focused on chronic MS pain in this population. The findings demonstrated that chronic MS pain was independently and significantly associated with hyperuricemia and the product of calcium and phosphate levels. In CKD patients with hyperuricemia, chronic MS pain was negatively, independently, and significantly associated with the presence of DM. However, in CKD patients without hyperuricemia, chronic MS pain was positively, independently, and significantly associated with the product of the calcium and phosphate levels. Furthermore, CKD stage 5 patients seemed to suffer from more severe chronic MS pain compared to other stage CKD patients.

These findings suggest that co-morbidity of hyperuricemia may be one of the major co-morbidities that contribute to chronic MS pain in CKD patients after adjustment of other confounders. This finding may account for the high association between hyperuricemia and CKD [18]. Hyperuricemia could be a consequence of impaired kidney function, diuretic therapy, or oxidative stress [19]. Therefore, it is not surprising that the co-morbidity of hyperuricemia is associated with chronic MS pain. However, the results demonstrated that there was no significant difference in uric acid levels between CKD patients with and without chronic MS pain. This finding may confirm that normal serum uric acid level at the onset of CKD does not exclude an acute gouty attack [20]. Adequate uric acid medical control for chronic MS pain patients with hyperuricemia may also explain the similar uric acid levels in the study patients with or without chronic MS pain.

Chronic pain may be caused by various factors. This study only focused on MS pain and excluded other origins of chronic pain, such as neuropathic pain. The possible sources of chronic MS pain are gout, renal bone disease, and ischemic bone pain. The results showed that the calcium × phosphate product levels were significantly associated with chronic MS pain. Renal bone disease is a common complication of CKD. Imbalance of calcium and phosphate, vitamin D deficiency, and hyperparathyroidism are considered to cause renal bone disease [21]. However, in this study, patients with chronic MS pain had similar serum calcium, phosphate, and iPTH levels as those patients without chronic MS pain. This suggests that renal bone disease may not be the cause of chronic MS pain in the CKD patients under study. The calcium × phosphate product levels had been found to correlate with vascular calcification [22]. Therefore, vascular calcification-related micro-angiopathy and ischemic bone pain may explain why this product was strongly associated with chronic MS pain, especially in patients without hyperuricemia. Golan et al. [11] found that higher serum calcium and iPTH levels were independently associated with chronic pain in haemodialysis patients. The differences in the study populations between CKD patients pre- and post-dialysis may explain these different findings. However, these results together suggest that the calcium-phosphate imbalance is a crucial factor for chronic MS pain in CKD patients.

Diabetic nephropathy is a common complication of diabetes mellitus. In the present study, it was found that 30.7% of patients had DM as co-morbidity. This study found that patients with chronic MS pain were less likely to have DM than those with no chronic MS pain (26.7% vs. 35.2%, Table 1). In multivariate analysis, it was found that the co-morbidity of DM was negatively associated with chronic MS pain in CKD patients with hyperuricemia but not in CKD patients without hyperuricemia. One possible explanation is that there is a negative association between co-morbidity of DM and hyperuricemia. However, in the present study it was found that there was no significant association between DM and hyperuricemia. Moreover, several studies have found high prevalence of gout in patients with Type 2 diabetes [23,24]. Choi et al. found that hyperuricemia is a risk factor for diabetes mellitus [25]. Moreover, Lai et al. found that gout and type 2 diabetes mellitus shared the most common genetic factors, which explains why there existed a mutual inter-dependent effect on higher incidences [26]. This may also be due to diabetic neuropathy. Previous studies have shown that pain-inhibiting neuropathy caused by glucose metabolism dysfunction may hide osteoarthritis pain and delay diagnosis of osteoarthritis [27]. Moreover, silent ischemia with less chest pain during acute coronary syndrome was significantly higher in diabetic patients, probably due to cardiac autonomic neuropathy [28]. Consequently, it is possible that diabetic neuropathy in diabetic CKD patients may inhibit or mask chronic MS pain. This may explain the finding of diabetes as a protective factor from chronic MS pain in CKD patients with hyperuricemia.

The study found that patients with chronic MS pain had a similar prevalence of NSAIDs or Chinese herbal medication use as those patients without chronic MS pain. In our study, there was no difference in use of NSAIDs or Chinese herbal medication between patients with hyperuricemia and without hyperuricemia. One possible explanation regarding the lack of association between NSAIDs use in CKD patients with pain may be due to our centre’s policy to reduce the use of NSAIDs in patients with chronic MS pain. Our CKD education provides CKD patients with information on protecting renal function, including better blood pressure control and avoidance of NSAIDs and Chinese herbal medication use. There was a significantly lower rate of NSAIDs use (7.8%) in our CKD study patients with chronic MS pain as compared to the report of Pham et al. (23.2%) [29]. This may be the reason why our study patients had lower prevalence of NSAIDs use and Chinese herbal medication. Another explanation is that CKD patients in nephrology clinics were usually reminded of avoiding NSAIDs agents in order to protect their kidney function.

The present study revealed that 53.3% patients had chronic MS pain, with a mean eGFR around 65 ml/min. However, Cohen et al. [10] reported that 69% of CKD patients with eGFR around 33.4 mL/min experience pain. The difference in results may be due to the definition of pain duration. The present study focused on chronic MS pain lasting for more than 3 months. However, Cohen et al. [10] defined pain duration on the basis of the pain experienced by the patients in the past month. Besides, the present study patients are 100% Asian, whereas 74% of Cohen’s patients were Afro-Americans. Thus, racial differences may also contribute to the difference in prevalence of pain in CKD patients. Nevertheless, these data provide information on the prevalence of chronic MS pain in CKD patients.

The results of the present study indicate a correlation between the co-morbidity of hyperuricemia, the calcium × phosphate product levels, and the co-morbidity of DM with chronic MS pain in CKD patients. This study raises the importance of uric acid control and gout prevention to decrease the possibility of chronic MS pain, thereby improving the quality of life in CKD patients. In addition, the study found that the calcium × phosphate product levels were an important factor for chronic MS pain, especially in CKD patients without hyperuricemia. Results of this study demonstrate that the calcium × phosphate product levels are an important laboratory parameter to consider while treating CKD patients with chronic MS pain and without hyperuricemia. Additionally, it was demonstrated that NSAID or Chinese herb use was not very common in CKD patients. Low prevalence of NSAID and Chinese herb use (7.8% and 9.6%) may indicate either good patient education by healthcare professionals, or an underestimation or inaccurate reporting of NSAID and Chinese herb use by patients.

This study had several limitations. The information regarding pain relief agents was lacking. Furthermore, the colchicine use for gout relief in study patients was not collected. In addition, quality of life was not evaluated in this study. However, we believe that the results provide a solid basis for studies that will further explore these relationships. The ultimate goal should be a better understanding and treatment of chronic MS pain in CKD patients.

## Conclusion

In summary, the present study demonstrated that chronic MS pain was independently and significantly associated with hyperuricemia and the calcium × phosphate product levels. In addition, in CKD patients with hyperuricemia, chronic MS pain was negatively, independently, and significantly associated with DM. However, in CKD patients without the co-morbidity of hyperuricemia, chronic MS pain was independently and significantly associated with the calcium × phosphate product levels.

## Competing interest

The authors declare no financial competing or other competing interest.

## Authors’ contributions

H-J H participated in data collection, statistical analysis and manuscript preparation; C-H Y, K-H H, and C-C L participated in statistical analysis; I-W W, M-J H, C-Y S, C-C C, Y-C C helped with data collection; M-F H, C-Y C, C-Y H, and C-J T participated in data interpretation; M-S W participated in study design, coordination, and helped to draft the manuscript. All authors read and approved the final manuscript.

## Authors’ information

Heng-Jung Hsu and Chiung-Hui Yen are Co-first authors.

## Pre-publication history

The pre-publication history for this paper can be accessed here:

http://www.biomedcentral.com/1471-2369/15/6/prepub
